# Supplementary material for: Importance of Self-Efficacy in Eating Behavior and Physical Activity Change of Overweight and Non-Overweight Adolescent Girls Participating in Healthy Me: A Lifestyle Intervention with Mobile Technology
Source: Nutrients. 2020 Jul 17;12(7):2128. doi: 10.3390/nu12072128 (PMC7400873; doi:10.3390/nu12072128)
Supplement: Supplementary file 1 [file nutrients-12-02128-s001.pdf]

Table S1. By the type of intervention and body weight status (%).

Intervention Group	Full						Partial						Null					
**Body Weight Status**	**Overweight**			**Non-Overweight**			**Overweight**			**Non-Overweight**			**Overweight**			**Non-Overweight**		
**Study**				**S1**	**S2**	**S3**	**S1**	**S2**	**S3**	**S1**	**S2**	**S3**	**S1**	**S2**	**S3**	**S1**	**S2**	**S3**
Fruit consumption																		
< once a week	8.5	9.2	4.2	3.8	4.7	4.9	6.8	5.1	3.4	7.4	7.4	5.3	5.3	7.0	8.6	6.4	5.9	4.5
Once a week	5.6	8.5	7.7	7.6	9.4	9.3	8.5	5.1	6.8	8.4	9.5	11.1	10.5	6.6	13.8	8.4	7.4	7.4
2–6 days a week	50.7	45.8	50.7	52.7	51.9	48.2	52.5	55.9	50.8	54.7	55.0	55.0	47.4	49.6	43.1	46.0	51.0	54.0
Daily	35.2	36.6	37.3	35.9	34.0	37.6	32.2	33.9	39.0	29.5	28.0	28.6	36.8	36.8	34.5	39.1	35.6	34.2
Vegetables consumption																		
< once a week	7.8	6.6	4.9	6.0	5.4	6.7	8.6	5.2	5.2	6.3	7.4	4.8	14.0	1.8	5.2	8.0	6.9	7.5
Once a week	8.5	5.1	9.9	10.7	8.7	8.2	5.2	12.1	13.8	10.1	8.5	7.4	5.3	12.5	8.6	7.5	7.4	6.5
2–6 days a week	44.7	52.6	50.7	46.2	50.4	51.4	44.8	56.9	46.6	52.9	54.0	60.1	49.1	44.6	46.6	46.3	50.0	52.2
Daily	39.0	35.8	34.5	37.1	35.4	33.7	41.4	25.9	34.5	30.7	30.2	27.7	31.6	41.1	39.7	38.3	35.6	33.8
Sweets consumption																		
Daily	18.3	14.5	11.3	30.5	21.2	19.6	15.5	13.6	10.3	36.0	28.0	24.7	8.8	16.4	8.6	31.8	19.9	19.9
2–6 days a week	47.2	47.8	49.6	49.4	50.7	50.3	50.0	59.3	34.5	42.9	50.8	48.4	52.6	41.8	41.4	48.8	59.2	52.7
Once a week	21.8	21.0	19.9	11.1	16.0	17.1	19.0	15.3	22.4	11.1	12.7	15.3	22.8	25.5	31.0	13.4	12.9	13.4
< once a week	12.7	16.7	19.1 [c]	8.9	12.1	13.0 [a]	15.5	11.9	32.8 [a]	10.1	8.5	11.6 [c]	15.8	16.4	19.0	6.0	8.0	13.9 [a]
Sugared drinks consumption																		
Daily	8.5	11.4	9.9	11.2	8.7	7.4	0.0	3.5	7.0	12.2	16.9	14.8	5.3	3.5	10.3	15.9	9.5	10.5
2–6 days a week	34.5	33.6	21.1	26.3	30.3	25.2	25.4	26.3	28.1	30.0	36.0	32.3	21.1	26.3	15.5	22.9	32.8	23.0
Once a week	14.1	16.4	14.8	15.8	15.5	18.1	23.7	15.8	17.5	17.6	12.7	20.1	19.3	15.8	22.4	15.4	10.0	22.0
< once a week	43.0	38.6	54.2 [b]	46.7	45.5	49.3 [c]	50.8	54.4	47.4 [b]	39.9	34.4	32.8 [b]	54.4	54.4	51.7	45.8	47.8	44.5
Breakfast weekdays																		
Never	21.1	23.2	20.4	19.3	20.9	17.7	16.9	20.3	11.9	25.9	33.2	31.7	27.6	26.3	25.9	20.4	23.8	18.3
1–2 days	8.5	16.2	10.6	9.3	11.1	12.6	10.2	11.9	13.6	15.3	16.8	12.2	15.5	8.8	6.9	11.9	13.4	13.4
3–4 days	17.6	10.6	16.2	16.7	18.0	19.3	16.9	18.6	16.9	11.1	13.2	10.6	12.1	12.3	17.2	14.4	12.4	18.3
Daily	52.8	50.0	52.8 [a]	54.7	50.0	50.3 [a]	55.9	49.2	57.6 [b]	47.6	36.8	45.5 [a]	44.8	52.6	50.0 [a]	53.2	50.5	50.0 [a]
Breakfast weekends																		
Never	5.6	5.0	7.8	2.4	5.1	4.3	1.8	3.4	3.4	2.4	4.8	6.3	1.8	5.3	3.4	5.0	5.0	5.0
1 day	14.8	17.9	16.3	8.4	11.9	13.2	17.5	22.4	20.3	8.4	15.9	22.1	10.5	10.5	8.6	11.0	13.5	19.4
Both days	79.6	77.1	75.9	89.1	83.0	82.5 [a]	80.7	74.1	76.3	89.1	79.4	71.6 [a]	87.7	84.2	87.9	84.0	81.5	75.6
MVPA [1]																		

never	6.6	4.9	6.4	4.2	4.0	6.7	5.1	3.4	10.2	5.8	8.4	8.5	1.7	0.0	3.4	2.0	3.0	4.5
1–3 days	36.0	38.7	41.4	31.1	39.5	42.3	54.2	46.6	44.1	39.7	41.1	51.3	46.6	47.4	51.7	36.1	35.3	36.6
4–6 days	44.1	41.5	45.0	49.1	41.2	41.4	35.6	39.7	35.6	43.9	41.1	33.3	46.6	47.4	41.4	47.0	48.3	52.0
7 days	13.2	14.8	7.1 [a]	15.6	15.3	9.6 [a]	5.1	10.3	10.2 [a]	10.6	9.5	6.9 [a]	5.2	5.3	3.4 [a]	14.9	13.4	6.9 [a]

Differences between 3 study periods by the Kendall's W test: [a] $p < 0.001$; [b] $p < 0.01$; [c] $p < 0.05$. [1]MVPA: moderate-to-vigorous physical activity.

Table S2. Health Behavior Index (HBI) change in 3 study periods by the type of intervention.

	**Type of Intervention**			p^2
	**Full**	**Partial**	**Null**	
	M ± SD	M ± SD	M ± SD	
Study 1	13.89 ± 3.17	13.24 ± 3.34	13.64 ± 3.21	0.033
Study 2	14.91 ± 4.07	13.88 ± 4.00	15.16 ± 4.19	<0.001
Study 3	13.93 ± 3.26	13.08 ± 3.42	13.83 ± 3.22	0.008
p^1	<0.001	0.001	<0.001	

[1] Differences in HBI between 3 study rounds—Kendall's W test for repeated measures. [2] Differences by type of intervention—H^2 Kruskal–Wallis test for independent groups.

Table S3. Changes in the self-efficacy before and after the Healthy Me program by the type of intervention.

	**Type of Intervention**			p^2
	**Full**	**Partial**	**Null**	
General Self-Efficacy (GSE)				
Study 1	35.04 ± 5.51	34.73 ± 5.04	33.83 ± 5.10	0.009
Study 3	34.56 ± 5.17	33.93 ± 5.43	34.15 ± 4.90	0.392
p^1	0.030	0.027	0.354	
Domain of strength				
Study 1	17.57 ± 3.11	17.46 ± 3.20	16.86 ± 3.23	0.009
Study 3	17.29 ± 3.13	17.07 ± 3.39	16.89 ± 3.28	0.342
p^1	0.020	0.138	0.832	
Domain of perseverance				
Study 1	17.40 ± 3.44	17.31 ± 3.07	16.99 ± 3.32	0.305
Study 3	17.26 ± 3.15	16.85 ± 3.17	17.22 ± 3.10	0.379
p^1	0.262	0.060	0.354	

[1] Differences in self-efficacy between 1st and 3rd study rounds—Kendall's W test for repeated measures.
[2] Differences by type of intervention—H^2 Kruskal–Wallis test for independent groups.
